# Supplementary material for: Dynamic three-sided matching model for personnel–robot-position matching problem in intelligent environments
Source: PLoS One. 2023 Apr 7;18(4):e0282312. doi: 10.1371/journal.pone.0282312 (PMC10081803; doi:10.1371/journal.pone.0282312)
Supplement: S1 File — (DOCX) [file pone.0282312.s001.docx]

**The following three tables, Table A1, Table A2 and Table A3, are the data assessed by experts.**

Table A.1 Evaluation matrix of personnel on intelligent robot

|  |  | $B_{1}$ | $B_{2}$ | $B_{3}$ | $B_{4}$ | $B_{5}$ |
| --- | --- | --- | --- | --- | --- | --- |
| T=1 | $A_{1}$ | [0.6,0.8] | [0.2,0.4] | [0.2,0.4] | [0.4,0.6] | [0.6,0.8] |
|  | $A_{2}$ | [0,0.2] | [0.2,0.4] | [0.4,0.6] | [0.8,1] | [0,0.2] |
|  | $A_{3}$ | [0.8,1] | [0.6,0.8] | [0.6,0.8] | [0.8,1] | [0.6,0.8] |
|  | $A_{4}$ | [0.6,0.8] | [0.8,1] | [0,0.2] | [0.4,0.6] | [0.6,0.8] |
|  | $A_{5}$ | [0.2,0.4] | [0.8,1] | [0.8,1] | [0.4,0.6] | [0.2,0.4] |
|  | $A_{6}$ | [0.8,1] | [0.8,1] | [0.2,0.4] | [0.4,0.6] | [0.8,1] |
| T=2 | $A_{1}$ | [0.4,0.6] | [0.2,0.4] | [0,0.2] | [0.4,0.6] | [0.4,0.6] |
|  | $A_{2}$ | [0,0.2] | [0.8,1] | [0.8,1] | [0,0.2] | [0,0.2] |
|  | $A_{3}$ | [0.8,1] | [0.4,0.6] | [0,0.2] | [0.4,0.6] | [0.8,1] |
|  | $A_{4}$ | [0.8,1] | [0.8,1] | [0,0.2] | [0,0.2] | [0.8,1] |
|  | $A_{5}$ | [0,0.2] | [0.8,1] | [0.2,0.4] | [0.8,1] | [0,0.2] |
|  | $A_{6}$ | [0.2,0.4] | [0.8,1] | [0.8,1] | [0.4,0.6] | [0.2,0.4] |
| T=3 | $A_{1}$ | [0.8,1] | [0.2,0.4] | [0.8,1] | [0.6,0.8] | [0.8,1] |
|  | $A_{2}$ | [0.6,0.8] | [0.2,0.4] | [0.4,0.6] | [0.6,0.8] | [0.8,1] |
|  | $A_{3}$ | [0.6,0.8] | [0.2,0.4] | [0,0.2] | [0.2,0.4] | [0.6,0.8] |
|  | $A_{4}$ | [0.8,1] | [0.2,0.4] | [0.4,0.6] | [0.6,0.8] | [0.6,0.8] |
|  | $A_{5}$ | [0.8,1] | [0.4,0.6] | [0,0.2] | [0.4,0.6] | [0.8,1] |
|  | $A_{6}$ | [0.8,1] | [0,0.2] | [0.8,1] | [0.8,1] | [0.2,0.4] |

Table A.2 Evaluation matrix of personnel to position

|  |  | $C_{1}$ | $C_{2}$ | $C_{3}$ | $C_{4}$ |
| --- | --- | --- | --- | --- | --- |
| T=1 | $A_{1}$ | [0,0.2] | [0.2,0.4] | [0.6,0.8] | [0.8,1] |
|  | $A_{2}$ | [0.8,1] | [0.8,1] | [0,0.2] | [0.6,0.8] |
|  | $A_{3}$ | [0.6,0.8] | [0.2,0.4] | [0.8,1] | [0.4,0.6] |
|  | $A_{4}$ | [0.4,0.6] | [0.2,0.4] | [0.6,0.8] | [0.8,1] |
|  | $A_{5}$ | [0.4,0.6] | [0.4,0.6] | [0.4,0.6] | [0,0.2] |
|  | $A_{6}$ | [0.6,0.8] | [0.4,0.6] | [0.4,0.6] | [0.6,0.8] |
| T=2 | $A_{1}$ | [0.4,0.6] | [0.2,0.4] | [0,0.2] | [0.4,0.6] |
|  | $A_{2}$ | [0,0.2] | [0.8,1] | [0.8,1] | [0,0.2] |
|  | $A_{3}$ | [0.8,1] | [0.4,0.6] | [0,0.2] | [0.4,0.6] |
|  | $A_{4}$ | [0.8,1] | [0.8,1] | [0,0.2] | [0,0.2] |
|  | $A_{5}$ | [0,0.2] | [0.8,1] | [0.2,0.4] | [0.8,1] |
|  | $A_{6}$ | [0.2,0.4] | [0.8,1] | [0.8,1] | [0.4,0.6] |
| T=3 | $A_{1}$ | [0,0.2] | [0.4,0.6] | [0.6,0.8] | [0,0.2] |
|  | $A_{2}$ | [0.4,0.6] | [0,0.2] | [0.6,0.8] | [0.4,0.6] |
|  | $A_{3}$ | [0.4,0.6] | [0.4,0.6] | [0.8,1] | [0.6,0.8] |
|  | $A_{4}$ | [0.6,0.8] | [0.8,1] | [0.8,1] | [0.8,1] |
|  | $A_{5}$ | [0.6,0.8] | [0.6,0.8] | [0.8,1] | [0.6,0.8] |
|  | $A_{6}$ | [0.8,1] | [0.8,1] | [0.2,0.4] | [0,0.2] |

Table A.3 Evaluation matrix of intelligent robot to position

|  |  | $C_{1}$ | $C_{2}$ | $C_{3}$ | $C_{4}$ |
| --- | --- | --- | --- | --- | --- |
| T=1 | $B_{1}$ | [0.8,1] | [0.4,0.6] | [0.2,0.4] | [0.8,1] |
|  | $B_{2}$ | [0.8,1] | [0.6,0.8] | [0.4,0.6] | [0.4,0.6] |
|  | $B_{3}$ | [0.8,1] | [0.4,0.6] | [0.8,1] | [0,0.2] |
|  | $B_{4}$ | [0.6,0.8] | [0.6,0.8] | [0,0.2] | [0.2,0.4] |
|  | $B_{5}$ | [0.2,0.4] | [0.6,0.8] | [0,0.2] | [0.4,0.6] |
| T=2 | $B_{1}$ | [0.2,0.4] | [0.8,1] | [0.8,1] | [0.8,1] |
|  | $B_{2}$ | [0.6,0.8] | [0.8,1] | [0.4,0.6] | [0.8,1] |
|  | $B_{3}$ | [0.6,0.8] | [0.8,1] | [0.4,0.6] | [0.4,0.6] |
|  | $B_{4}$ | [0.6,0.8] | [0.8,1] | [0.8,1] | [0.4,0.6] |
|  | $B_{5}$ | [0,0.2] | [0.2,0.4] | [0.2,0.4] | [0,0.2] |
| T=3 | $B_{1}$ | [0.8,1] | [0.2,0.4] | [0.6,0.8] | [0.2,0.4] |
|  | $B_{2}$ | [0.4,0.6] | [0.4,0.6] | [0.2,0.4] | [0.4,0.6] |
|  | $B_{3}$ | [0.4,0.6] | [0,0.2] | [0.8,1] | [0.6,0.8] |
|  | $B_{4}$ | [0,0.2] | [0.6,0.8] | [0.8,1] | [0.8,1] |
|  | $B_{5}$ | [0,0.2] | [0.2,0.4] | [0,0.2] | [0.4,0.6] |

Table A.4 Prospect value of personnel on intelligent robot

|  |  | $B_{1}$ | $B_{2}$ | $B_{3}$ | $B_{4}$ | $B_{5}$ |
| --- | --- | --- | --- | --- | --- | --- |
| T=1 | $A_{1}$ | 0.075 | -0.533 | -0.533 | -0.153 | 0.075 |
|  | $A_{2}$ | -0.992 | -0.533 | -0.153 | 0.452 | -0.992 |
|  | $A_{3}$ | 0.249 | 0.075 | 0.075 | 0.452 | 0.075 |
|  | $A_{4}$ | 0.075 | 0.452 | -0.992 | -0.153 | 0.075 |
|  | $A_{5}$ | -0.533 | 0.452 | 0.452 | -0.153 | -0.533 |
|  | $A_{6}$ | 0.249 | 0.452 | -0.533 | -0.153 | 0.452 |
| T=2 | $A_{1}$ | 0.065 | -0.177 | 0.436 | 0.436 | 0.065 |
|  | $A_{2}$ | 0.231 | 0.065 | 0.436 | -0.177 | 0.436 |
|  | $A_{3}$ | 0.231 | -1.029 | -0.177 | -0.572 | 0.436 |
|  | $A_{4}$ | 0.231 | 0.065 | -1.029 | -0.572 | -1.029 |
|  | $A_{5}$ | 0.065 | -0.177 | 0.065 | 0.436 | -0.177 |
|  | $A_{6}$ | 0.231 | -1.029 | -0.177 | 0.436 | -0.177 |
| T=3 | $A_{1}$ | 0.445 | -0.549 | 0.445 | 0.071 | 0.445 |
|  | $A_{2}$ | 0.071 | -0.549 | -0.163 | 0.071 | 0.445 |
|  | $A_{3}$ | 0.071 | -0.549 | -1.007 | -0.549 | 0.071 |
|  | $A_{4}$ | 0.241 | -0.549 | -0.163 | 0.071 | 0.071 |
|  | $A_{5}$ | 0.241 | -0.163 | -1.007 | -0.163 | 0.445 |
|  | $A_{6}$ | 0.241 | -1.007 | 0.445 | 0.445 | -0.549 |

Table A.5 Prospect value of personnel to position

|  |  | $C_{1}$ | $C_{2}$ | $C_{3}$ | $C_{4}$ |
| --- | --- | --- | --- | --- | --- |
| T=1 | $A_{1}$ | -0.909 | -0.441 | 0.098 | 0.489 |
|  | $A_{2}$ | 0.288 | 0.288 | -0.909 | 0.098 |
|  | $A_{3}$ | 0.098 | -0.441 | 0.288 | -0.098 |
|  | $A_{4}$ | -0.098 | -0.441 | 0.098 | 0.288 |
|  | $A_{5}$ | -0.098 | -0.098 | -0.098 | -0.909 |
|  | $A_{6}$ | 0.098 | -0.098 | -0.098 | 0.098 |
| T=2 | $A_{1}$ | -0.059 | -0.379 | -0.853 | -0.059 |
|  | $A_{2}$ | -0.853 | 0.314 | 0.314 | -0.853 |
|  | $A_{3}$ | 0.314 | -0.059 | -0.853 | -0.059 |
|  | $A_{4}$ | 0.314 | 0.314 | -0.853 | -0.853 |
|  | $A_{5}$ | -0.853 | 0.314 | -0.379 | 0.314 |
|  | $A_{6}$ | -0.379 | 0.314 | 0.314 | -0.059 |
| T=3 | $A_{1}$ | -0.955 | -0.129 | 0.085 | -0.955 |
|  | $A_{2}$ | -0.129 | -0.955 | 0.085 | -0.129 |
|  | $A_{3}$ | -0.129 | -0.129 | 0.266 | 0.085 |
|  | $A_{4}$ | 0.085 | 0.266 | 0.266 | 0.266 |
|  | $A_{5}$ | 0.085 | 0.085 | 0.266 | 0.085 |
|  | $A_{6}$ | 0.266 | 0.266 | -0.492 | -0.955 |

Table A.6 Prospect value of intelligent robot to position

|  |  | $C_{1}$ | $C_{2}$ | $C_{3}$ | $C_{4}$ |
| --- | --- | --- | --- | --- | --- |
| T=1 | $B_{1}$ | -0.909 | -0.441 | 0.098 | 0.489 |
|  | $B_{2}$ | 0.288 | 0.288 | -0.909 | 0.098 |
|  | $B_{3}$ | 0.098 | -0.441 | 0.288 | -0.098 |
|  | $B_{4}$ | -0.098 | -0.441 | 0.098 | 0.288 |
|  | $B_{5}$ | -0.098 | -0.098 | -0.098 | -0.909 |
| T=2 | $B_{1}$ | -0.514 | 0.460 | 0.460 | 0.460 |
|  | $B_{2}$ | 0.080 | 0.460 | -0.142 | 0.460 |
|  | $B_{3}$ | 0.080 | 0.460 | -0.142 | -0.142 |
|  | $B_{4}$ | 0.080 | 0.460 | 0.460 | -0.142 |
|  | $B_{5}$ | -0.975 | -0.514 | -0.514 | -0.975 |
| T=3 | $B_{1}$ | 0.523 | -0.352 | 0.119 | -0.352 |
|  | $B_{2}$ | -0.042 | -0.042 | -0.352 | -0.042 |
|  | $B_{3}$ | -0.042 | -0.829 | 0.523 | 0.119 |
|  | $B_{4}$ | -0.829 | 0.119 | 0.523 | 0.523 |
|  | $B_{5}$ | -0.829 | -0.352 | -0.829 | -0.042 |

**The code executed by solving the model.**

model:

sets:

worker/1 2 3 4 5 6/;

machine/1 2 3 4 5/;

position/1 2 3 4/;

manyidu(worker, machine,position):x;

linka(worker,machine):w;

linkb(machine,position):p;

linkc(worker,position):m;

endsets

[obj] MAX=@sum(worker(i):@sum(machine(j):@sum(position(k):x(i,j,k)*(0.5*w(i,j)+0.25*m(i,k)+0.25*p(j,k)))));

data:

w=

| 0.308 | -0.451 | 0.340 | 0.142 | 0.308 |
| --- | --- | --- | --- | --- |
| 0.000 | -0.388 | -0.007 | 0.047 | 0.292 |
| 0.131 | -0.607 | -0.679 | -0.450 | 0.166 |
| 0.221 | -0.285 | -0.474 | -0.119 | -0.213 |
| 0.114 | -0.102 | -0.576 | -0.007 | 0.182 |
| 0.239 | -0.859 | 0.182 | 0.380 | -0.347 |

;

m=

| -0.719 | -0.226 | -0.156 | -0.572 |
| --- | --- | --- | --- |
| -0.272 | -0.496 | 0.040 | -0.292 |
| 0.009 | -0.144 | -0.021 | 0.029 |
| 0.125 | 0.204 | -0.041 | -0.021 |
| -0.176 | 0.125 | 0.061 | 0.040 |
| 0.082 | 0.240 | -0.242 | -0.613 |

;

p=

| 0.252 | -0.115 | 0.150 | -0.053 |
| --- | --- | --- | --- |
| 0.046 | 0.103 | -0.270 | 0.083 |
| 0.046 | -0.418 | 0.348 | -0.055 |
| -0.496 | 0.205 | 0.357 | 0.251 |
| -0.825 | -0.346 | -0.755 | -0.288 |

;

enddata

@for(worker(i):

@sum(machine(j):

@sum(manyidu(i,j,k):x(i,j,k)))<=1);

@for(machine(j):

@sum(position(k):

@sum(manyidu(i,j,k):x(i,j,k)))<=1);

@for(position(k):

@sum(worker(i):

@sum(manyidu(i,j,k):x(i,j,k)))=1);

@for(manyidu(i,j,k):@bin(x(i,j,k)));

end
