# Supplementary material for: Dynamic three-sided matching model for personnel–robot-position matching problem in intelligent environments
Source: PLoS One. 2023 Apr 7;18(4):e0282312. doi: 10.1371/journal.pone.0282312 (PMC10081803; doi:10.1371/journal.pone.0282312)
Supplement: Attachment — Submitted filename: PONE-D-22-06663-comments.pdf [file pone.0282312.s002.pdf]

This paper proposed a dynamic three-sided decision model and applied it in a personnel–robot-position matching (PRPM) problem, which is an interesting topic. However, this paper should be revised carefully. Below are the comments:

1) It is not appropriate to use the abbreviation PRPM in paper title.

2) In the introduction, the motivation for using the prospect theory is unclear. Could authors provide more discussion about the relater research of PRPM problem?

3) The third paragraph of the introduction discusses two-sided matching researches, but they are mistakenly written as three-sided matching.

4) Please check the citation format in the main body and reference list of this paper. Especially the authors' name and the Chinese citations. Some page numbers are missing in the reference list.

5) Please check the notations of this paper. In Section 2, what is the range of N? What is the meaning of M? In Section 3, the risk aversion coefficient and the attenuation coefficient use the same symbol. In page 9, the definition 4 should be definition 5, and the symbols are missed.

6) The reference list should be updated, since many papers cited in the paper are quite old and not international journal papers, which is inappropriate for readers outside China. I suggest the authors add more papers about matching decision making published in recent years, for instance, Consistency improvement for fuzzy preference relations with self-confidence: An application in two-sided matching decision making; Two-sided matching decision making with multi-granular hesitant fuzzy linguistic term sets and incomplete criteria weight information. Please include and comment them in your paper.

7) In Section 3.1, the language preference information should be linguistic preference information. In page 7, for the third case, it is likely that the part [d^-,r^-] could be more than [d^+,r^+]. How to deal with this case?

8) It would be better if authors can provide some comparative analysis to justify the model.

9) The language of this paper should be improved. Please proofread the whole paper to avoid any grammar and syntax issues. I think a certification about language editing from a copyeditor is needed.
