# Supplementary material for: Dynamic three-sided matching model for personnel–robot-position matching problem in intelligent environments
Source: PLoS One. 2023 Apr 7;18(4):e0282312. doi: 10.1371/journal.pone.0282312 (PMC10081803; doi:10.1371/journal.pone.0282312)
Supplement: Attachment — Submitted filename: PLOSONE-response to reviewers-0810.docx [file pone.0282312.s003.docx]

**Dear editor,**

The authors are very grateful for the editor's editing work and the reviewer's suggestions to improve the manuscript and the responses to the reviewer's comments are given as follows.

**Reviewers' comments:**

**Reviewer #1:** This paper proposed a dynamic three-sided decision model and applied it in personnel–robot-position matching (PRPM) problem, which is an interesting topic. However, this paper should be revised carefully. My comments are attached.

1. It is not appropriate to use the abbreviation PRPM in paper title.

**Response:** The authors thank the reviewer's suggestions to improve the manuscript. The new title of the manuscript is determined as 'Dynamic three-sided matching model for personnel–robot-position matching problem in intelligent environment'.

2. In the introduction, the motivation for using the prospect theory is unclear. Could authors provide more discussion about the relater research of PRPM problem?

**Response:** The relevant discussions on PRPM issues are relatively weak and need to be further supplemented. The revised contents are as follows:

**Section 1, paragraph 1:** This paragraph puts forward the problem of this paper-PRPM.

**Section 1, paragraph 2:** The purpose of solving PRPM is to realize man-machine collaborative configuration. And the relevant research on PRPM is given. Points out the shortcomings of existing research: *Based on the studies, we find these studies focuses on the micro level, and does not face the entire manufacturing system. Another part of the studies considered the skill level of personnel from the perspective of the whole manufacturing system, but did not consider the factors such as safety and acceptability, and did not integrate the people-oriented manufacturing concept.*

**Section 1, paragraph 3:** In view of the above shortcomings, it is proposed that PRPM can be regarded as a three-sided matching problem. The research on three-sided matching has been carried out in many fields. **Section 1, paragraph 3, last sentence:** *However, in the matching decision-making process, the single-stage preference information can not fully reflect the preference degree of the matching subject, so most manufacturing enterprises use multi-stage evaluation. For the existing studies, the three-sided matching method is still static, ignoring the dynamic nature of multi-stage preference information.*

**Section 1, paragraph 4:** *This method comprehensively considers the psychological factors of decision makers and reflects the impact of preference information in different stages on the final decision-making results. Therefore, in this paper, prospect theory and attenuation index model are introduced to integrate the multi-stage preference information.*

3. The third paragraph of the introduction discusses two-sided matching researches, but they are mistakenly written as three-sided matching.

**Response:** Thank the reviewers for suggestions to improve the quality of the manuscript. We have made important changes to the introduction of the article. This part has been deleted. As shown in the reply to the previous proposal, we have readjusted the contents of paragraphs 1 to 4.

4. Please check the citation format in the main body and reference list of this paper. Especially the author’s name and the Chinese citations. Some page numbers are missing in the reference list.

**Response:** This suggestion is very valuable, and the standard format is an important prerequisite for ensuring high-quality papers. We have checked and revised the grammar, charts and formulas of the full text according to the requirements of PLOS One.

5. Please check the notations of this paper. In Section 2, what is the range of N? What is the meaning of M? In Section3, the risk aversion coefficient and the attenuation coefficient use the same symbol. In page 9, the definition 4 should be definition 5, and symbols are missed.

**Response:** We are sorry for the errors in details. We have made timely corrections. *The set composed of all matching groups is called a match of the matching system, and is set as M.* *Let* $N\left(M,x\right)$ *indicates the number of matching groups.*

**Section 3.4, paragraph 3:**$\rho$ is called risk aversion coefficient. The coding sequence number of definition 4 has been corrected.

6. The reference list should be updated, since many papers cited in the paper are quite old and not international journal papers, which is inappropriate for readers outside China. I suggest the authors add more papers about matching decision making published in recent years, for instance, Consistency improvement for fuzzy preference relations with self-confidence: An application in two-sided matching decision making; Two-sided matching decision making with multi-granular hesitant fuzzy linguistic term sets and incomplete criteria weight information. Please include and comment them in your paper.

**Response:** Thank the reviewers for their suggestions. The modification details are as follows:

In the second paragraph of the first section, we are sorted out the research on human-machine collaborative configuration, and supplemented a certain number of foreign literature to align with international journals. In the third paragraph of the first section, the application research of trilateral matching is reviewed.

7. In Section 3.1, the language preference information should be linguistic preference information. In page 7, for the third case, it is likely that the part {} could be more than []. How to deal with this case?

**Response:** linguistic preference information has replaced language preference information. We regret that the second point of the proposal was not well understood. In this paper, we fully consider six different quantitative relationships between preference values and dynamic reference points. In the third case, the lower limit value of the reference point is in the middle of the preference value, and the upper limit value of the reference point is greater than the upper limit value of the preference value, so the preference value and the dynamic reference point belong to a cross relationship.

8. It would be better if authors can provide some comparative analysis to justify the model.

**Response:** This is a very good suggestion. In the part of discussion and analysis, this paper discusses the objective function parameter analysis and the influence of attenuation index on the matching results. This is a self-comparison analysis method, but no better comparison method has been found to compare the proposed model horizontally. It can only be analyzed and verified from the actual cases proposed in this paper. In future, we are trying hard to do some comparative analysis to justify the model.

9. The language of this paper should be improved. Please proofread the whole paper to avoid any grammar and syntax issues. I think a certification about language editing from a copyeditor is need.

**Response:** The manuscript was revised with the help of a professional English editor.

R2

1. This paper designs a dynamic three-sided matching decision-making approach focusing on the three sides matching problem. I am confused that whether this problem is NP-hardness? If yes, whether the proposed algorithm can be completed within polynomial time? Then, what’s the approximate ratio of the output result?

**Response:** Thank the reviewers for their suggestions. The problem in this paper does not belong to NP problem.

**Section 5, paragraph 3:** *Obviously, the model (40)-(44) is a classical model for assignment problems. Simultaneously, the proposed problem is n×n×n assignment one. Some polynomial time algorithms have been proposed to solve the classical assignment problems, such as the Kuhn–Munkres algorithm and the Bertsekas algorithm. Further, the existing assignment algorithms can be used to solve the model (18)–(22) conveniently. On the other side, this proposed model can also be solved by applying the mathematical optimization software (e.g., LINGO 11.0).*

2. There are lots of methods based on dynamic matching theory introduced in the introduction of this paper, so I am curious that what’s the biggest improvement from your proposition over these works? Is it just a simple generalization from these work to your work?

**Response:** This is a very valuable suggestion. Compared with other studies, this paper proposes a new solution to the human-machine configuration problem in the intelligent environment - the combination of trilateral matching theory and fuzzy sets. The contribution in theory is to propose a multi-stage dynamic trilateral matching method. Inspired by this, we rearrange the research issues of this paper. We put forward a new solution to the problem of human-machine collaborative configuration in a highly intelligent environment. Aiming at the dynamic trilateral matching problem based on multi-stage preference information in human-machine cooperation mode, a multi-stage dynamic trilateral matching method is proposed. This method takes into account the psychology of decision makers and the changing trend of preference information of trilateral matching subjects in different stages, and realizes multi-stage dynamic decision-making.

3. More importantly, the contributions of this work are not very clear, so I think authors need to highlight their innovations in the introduction section.

**Response:** In view of the above suggestions, the following supplements are made in the introduction:

The main contributions of this paper are as follows:

1.	A scientific and systematic man-machine collaborative fuzzy configuration method is established to solve the problem of man-machine collaborative configuration in flexible assembly manufacturing system.

2.	The proposed method takes into account the psychology of decision makers and the changing trend of preference information of trilateral matching subjects in different stages, and realizes multi-stage dynamic decision-making.

4. In the simulation, whether an algorithm is needed as a benchmark to be compared with your algorithm? And a recurrent learning process is required to enhance the accuracy of the output? If not, how to guarantee the matching accuracy of your algorithm?

**Response:** Thank the reviewers for their suggestions. Lingo is the abbreviation of linear interactive and general optimizer, i.e., "interactive linear and general optimization solver". It can be used to solve nonlinear programming, and can be used to solve some linear and nonlinear equations. It has very powerful functions and is the best choice for solving optimization models. It is characterized by the built-in modeling language, which provides more than ten internal functions, and allows the decision variables to be integers (i.e., integer programming, including 0-1 integer programming), which is convenient and flexible, and the execution speed is very fast. In linear integer models, lingo will do a lot of pre-processing work before solving (such as "cutting" of constraints to limit the feasible non integer regions). These methods will greatly improve the speed and accuracy of most integer solving models.

In Section 5, we added pseudo code of model solving algorithm. Because the model proposed in this paper is a three-dimensional 0-1 assignment problem, and lingo software is used to solve the linear programming model proposed in this paper, the matching results can be obtained quickly and accurately. Specific supplementary contents are as follows:

As aforementioned, the matching algorithm is designed to address the proposed model. Specifically, the pseudo-codes of this proposed two-sided matching model can be summarized below:

MODEL:

SETS:

*A/1…m/;*

*B/1…n/;*

*C/1…l/;*

LINK*(A,B,C):α,β,γ;*

END SET

DATA: THE MATCHING MATRIX:

*α=[α_ij_]_m×n_,β=[β_ik_]_m×l_,γ=[γ_jk_]_n×l_*;

END DATA

MAX=@SUM(LINK: *α***X+β***X+γ***X*);

@FOR(A(I):@SUM(A(I):X(I,J,K))<=1);

@FOR(B(J):@SUM(B(J):X(I,J,K))<=1);

@FOR(B(J):@SUM(C(L):X(I,J,K))=1);

@FOR (LINK:@BIN(X));

END
