# Supplementary material for: Dynamic three-sided matching model for personnel–robot-position matching problem in intelligent environments
Source: PLoS One. 2023 Apr 7;18(4):e0282312. doi: 10.1371/journal.pone.0282312 (PMC10081803; doi:10.1371/journal.pone.0282312)
Supplement: Attachment — Submitted filename: renamed_0d5f7.docx [file pone.0282312.s004.docx]

**Dear editor,**

The authors are very grateful for the editor's editing work and the reviewer's suggestions to improve the manuscript and the responses to the reviewer's comments are given as follows.

**Reviewers' comments:**

**Reviewer #1:** Although some revisions have been made, there are still many problems that prevent the acceptcance of this paper. The authors should further revise the paper based on the following comments:

**1.** The reference list are not updated. I still find many papers cited in the paper are quite old and not international journal papers. The authors do not provide a good response to my previous comment #6. Please carefully revise the reference list by following my previous comments.

**Response:** First of all, I would like to thank the review experts for giving us the opportunity to revise again. In response to this problem, I conducted a literature survey again and added more excellent literature to support this study and the revised result is as follows:

**Section 1 paragraph 1: “***The two-sided matching theory has been applied in different fields, e.g. the matching problem between knowledge suppliers and knowledge demanders.”* and “*matching the supply and demand of green building technology*.” In view of the sixth comment in the first amendment proposal, we have supplemented the two literatures respectively. Because before introducing the trilateral matching theory, we need to introduce the research related to bilateral matching as a basic introduction and explanation. **See reference 10 and11.**

**Section 1 paragraph 4: “***Certain studies also proposed an incomplete fuzzy preference relationship based on the disappointment theory to represent the comparative preference information of other matching objects. The aim was to describe the disappointment and elation among the matching objects”* In further literature research, this view can be used as an important support for introducing decision-makers to consider psychological factors.

**Section 1 paragraph 4: “***The proposed method also has certain limitations. Multi-granular HFLTSs are generally elicited by decision makers in a multi-attribute group decision-making (MAGDM) problem owing to the uncertainty of the decision environment and differences in decision makers’ culture and knowledge background”* In the discussion and analysis part, this article has given us a lot of inspiration, so we have quoted it: *Consensus reaching for MAGDM with multi-granular hesitant fuzzy linguistic term sets: a minimum adjustment-based approach*. How to successfully apply a new fuzzy language to dynamic trilateral matching will be my new research point in the future.

2.Again, my previous comment #4 is not well addressed. There are many problems for the format of the reference list. The author’s name are not well displayed.

**Response:** The references format was revised. See the manuscript literature for details. For example:

9.	Liang, Z.-C., Yang, Y., and Liao, S.-G. (2022). Interval-valued intuitionistic fuzzy two-sided matching model considering level of automation*.* Applied Soft Computing. **116**, 108252.

10.	Zhang, Z., Kou, X., Yu, W., and Gao, Y. (2020). Consistency improvement for fuzzy preference relations with self-confidence: An application in two-sided matching decision making*.* Journal of the Operational Research Society, 1-14.

3. If you cannot provide numerical comparative studies, you can provide some discussions through comparing with exsiting studies. Comprarative studies are missing.

**Response:** Thank the reviewers for their suggestions to help us further improve our paper. In this regard, we are inspired by the literature: " ***Extended TODIM for multi-criteria group decision making based on unbalanced hesitant fuzzy linguistic term sets*** ". We discussed and analyzed from three dimensions. The details are as follows:

1) First, the matching results of the model are observed by adjusting the weights of the three objective functions. In this case, the designed weights are 0.5, 0.25, and 0.25, respectively. Therefore, we redesign different weight coefficients to observe the calculation results (see Table 6).
2) Second, the influence of the attenuation coefficient on matching is analysed. The attenuation coefficient is the key parameter affecting the final satisfaction matrix. It is necessary to further examine the results of dynamic trilateral matching under different attenuation coefficients.
3) Third, we discuss the advantages and disadvantages of the methods proposed in this paper from a theoretical perspective and compared with different references.

4. The language is still quite poor. Please ask a copyeditor to improve the language and a certification is needed.

**Response:** The manuscript has been edited by professional editors. The modification certificate is as follows:

**Reviewer #2:** The demonstration of the pseudo-codes of the proposed algorithm is not very standard, so please revise it in a more standard format. Authors have solved all my given comments, so this paper can be accepted for publication in this journal.

**Response:** Thank the experts for their affirmation. The pseudo code of the algorithm is normalized, and the modification results are as follows:

1: MODEL:

2: SETS:

*3: A/1…m/;*

*4: B/1…n/;*

*5: C/1…l/;*

6: LINK*(A,B,C):α,β,γ;*

7: END SET

8: DATA: THE MATCHING MATRIX:

*9: α=[α_ij_]_m×n_,β=[β_ik_]_m×l_,γ=[γ_jk_]_n×l_*;

10: END DATA

11: MAX=@SUM(LINK: *α***X+β***X+γ***X*);

12: @FOR(A(I):@SUM(A(I):X(I,J,K))<=1);

13: @FOR(B(J):@SUM(B(J):X(I,J,K))<=1);

14: @FOR(B(J):@SUM(C(L):X(I,J,K))=1);

15: @FOR (LINK:@BIN(X));

16: END

**The following is the execution code of this program:**

model:

sets:

worker/1 2 3 4 5 6/;

machine/1 2 3 4 5/;

position/1 2 3 4/;

manyidu(worker, machine,position):x;

linka(worker,machine):w;

linkb(machine,position):p;

linkc(worker,position):m;

endsets

[obj] MAX=@sum(worker(i):@sum(machine(j):@sum(position(k):x(i,j,k)*(0.5*w(i,j)+0.25*m(i,k)+0.25*p(j,k)))));

data:

w=

0.308	-0.451	0.340	0.142	0.308

0.000	-0.388	-0.007	0.047	0.292

0.131	-0.607	-0.679	-0.450	0.166

0.221	-0.285	-0.474	-0.119	-0.213

0.114	-0.102	-0.576	-0.007	0.182

0.239	-0.859	0.182	0.380	-0.347

;

m=

-0.719	-0.226	-0.156	-0.572

-0.272	-0.496	0.040	-0.292

0.009	-0.144	-0.021	0.029

0.125	0.204	-0.041	-0.021

-0.176	0.125	0.061	0.040

0.082	0.240	-0.242	-0.613

;

p=

0.252	-0.115	0.150	-0.053

0.046	0.103	-0.270	0.083

0.046	-0.418	0.348	-0.055

-0.496	0.205	0.357	0.251

-0.825	-0.346	-0.755	-0.288

;

enddata

@for(worker(i):

@sum(machine(j):

@sum(manyidu(i,j,k):x(i,j,k)))<=1);

@for(machine(j):

@sum(position(k):

@sum(manyidu(i,j,k):x(i,j,k)))<=1);

@for(position(k):

@sum(worker(i):

@sum(manyidu(i,j,k):x(i,j,k)))=1);

@for(manyidu(i,j,k):@bin(x(i,j,k)));

end

Finally, the authors thank the reviewer again for the useful comments and all my best wishes.
