# Supplementary material for: Dynamic three-sided matching model for personnel–robot-position matching problem in intelligent environments
Source: PLoS One. 2023 Apr 7;18(4):e0282312. doi: 10.1371/journal.pone.0282312 (PMC10081803; doi:10.1371/journal.pone.0282312)
Supplement: Attachment — Submitted filename: Response to Reviewers-20230106.docx [file pone.0282312.s005.docx]

**Dear editor,**

The authors are very grateful for the editor's editing work and the reviewer's suggestions to improve the manuscript and the responses to the reviewer's comments are given as follows.

**Reviewers' comments:**

Reviewer #2: This paper establishes a dynamic three-sided matching model striving to maximize the matching satisfaction of multiple sides. My reviewing comments are listed as follows:

1. English presentation needs to be improved significantly.

**Response:** The manuscripts were rechecked and proofread for language, grammar, phrase, clarity, and readability with the professional help of the Elsevier Institute.

2. The layout of the paper is not particularly graceful, as the demonstration of tables, figures and formulas is not very standard.

**Response:** Revise charts, formulas, etc. in strict accordance with the requirements. Figure 4 shows the results of the LINGO software calculation model. After the consensus of the authors, it is unnecessary to put the screenshot of the LINGO software calculation results in the text, because there is no more important information except the maximum objective function value.

3. It's not clear that how to use the fuzzy language to quantify the uncertain information characteristics, and whether to apply a fuzzy function and the if-then rule. Besides, some references focusing on the fuzzy method in intelligent environment are not included in the paper, such as

K. Liu et al., "Big Medical Data Decision-Making Intelligent System Exploiting Fuzzy Inference Logic for Prostate Cancer in Developing Countries," in IEEE Access, vol. 7, pp. 2348-2363, 2019, doi: 10.1109/ACCESS.2018.2886198 Add to Citavi project by DOI.

**Response:** The advantage of the fuzzy language used in the manuscript is that experts can quickly give the matching degree between matching subjects according to experience. See Section 3.1 for details. Secondly, the literature Big Medical Data Decision Making Intelligent System Exploring Fuzzy Input Logic forState Cancer in Developing Countries has been added in the third paragraph of Section 1: *Obvious, fuzzy sets have been widely used in the three sided matching problem. The fuzzy set theory has also been successfully applied in the Big medical data decision-making intelligent system to observe the curative powers of the deterministic treatment method on patients in real time[27]*.

4. The representativeness of the given case study is missing. Please add it.

**Response: SECTION6:** *During the construction of the production line, it is expected that the operators will include mature employees and newly recruited employees from other production lines, and the robots will include newly purchased robots of various models and old robots retained for transformation. Therefore, the constructors of production lines need to evaluate the operators and robots, and identify the key stations, to put high-quality and suitable robots and operators into the key stations.*

*C_1_ position completes part of the assembly tasks in the rear floor area of the car body. The work content of this station includes high-strength structural parts of the car body, and its dimensional accuracy and stability are poor. C_2_ position and C_3_ position are a pair of left and right mirror stations, which complete part of the assembly tasks on the right and left side of the body side wall. The work involves many parts, and has a great impact on the assembly of left and right doors, cockpit and other performance. C_4_ position is C_2_ position next to the previous position, and there are relatively few assembly parts. In structure, it is next to the operating parts of C_1_ position, so the operation content of this position has a great impact on the function.*

*In our study, preference information used in the experiment is acquired from an automobile manufacturer in Chongqing. Furthermore, the information recording centre collects, classifies, pre-processes, and integrates various matching subjects based on different stages of manufacturing. These statistics mainly reflect the positions, intelligent robots, and employees over the entire manufacturing life cycle.*

5. The pseudo-code presentation is not very standard, especially lacking the inputs and outputs.

**Response:** The writing method of pseudo code in page 11 of reference [27], and modify the code as required.

Finally, the authors thank the reviewer again for the useful comments and all my best wishes.
